# Role of gut microbiota and inflammatory factors in acute respiratory distress syndrome: a Mendelian randomization analysis

**DOI:** 10.3389/fmicb.2023.1294692

**Published:** 2023-12-20

**Authors:** Jiawei Ma, Zigang Zhu, Yisikandeer Yishajiang, Khaloud Mohammed Alarjani, Lei Hong, Liang Luo

**Affiliations:** ^1^Department of Critical Care Medicine, Jiangnan University Medical Center, Wuxi, China; ^2^Department of Critical Care Medicine, Aheqi County People's Hospital, Xinjiang, China; ^3^Department of Botany and Microbiology, College of Science, King Saud University, Riyadh, Saudi Arabia; ^4^Institute of Clinical Medicine Research, The Affiliated Suzhou Science and Technology Town Hospital of Nanjing Medical University, Suzhou, China

**Keywords:** acute respiratory distress syndrome, gut microbiota, inflammatory factors, Mendelian randomization, genome-wide association studies

## Abstract

**Background:**

Acute respiratory distress syndrome (ARDS) is a serious lung ailment marked by significant inflammation and damage in the alveoli and capillaries of the lungs. Recent research suggests a strong correlation between the onset and advancement of ARDS and an imbalance in the gut microbiota (GM).

**Methods:**

In this investigation, Mendelian randomization (MR) analysis was utilized, drawing on data from publicly accessible genome-wide association studies. The primary focus was on examining the interplay between GM, inflammatory factors (IFs) and ARDS. Instrumental variables were established through genetic modifications of GM and IFs. Various statistical analysis methods including the inverse-variance weighted model, MR-Egger method and Wald ratio test were applied for comprehensive data analysis.

**Results:**

Eight bacterial taxa within the GM demonstrated a potential causal link with development of ARDS. Notably, the phylum Actinobacteria and the genus *Intestinibacter* exhibited a negative association with the risk of ARDS. However, *Erysipelotrichales* (id. 2,148), *Victivallis* (id. 2,256), *Ruminococcaceae* UCG014 (id. 11,371), *Eubacterium ruminantium* group (id. 11,340), *Erysipelotrichaceae* (id. 2,149) and *Erysipelotrichia* (id. 2,147) demonstrated a positive association with ARDS risk. Additionally, the study identified a potential causal relationship between the inflammatory factors interleukin-16 and C-C motif chemokine 3 with the occurrence of ARDS.

**Conclusion:**

This study strongly suggests that the interaction between gut microbiota (GM) and inflammatory factors (IFs) significantly contributes to the pathogenesis of acute respiratory distress syndrome (ARDS). This underscores their crucial involvement in both the initiation and advancement of this severe lung disorder.

## 1 Introduction

Acute respiratory distress syndrome (ARDS) is a severe pulmonary condition characterized by pronounced lung inflammation and increased permeability of pulmonary blood vessels resulting in respiratory failure, diminished oxygen levels and observable opacities in lung radiographs (Yıldırım et al., 2021; Fujishima, 2023). The etiology of ARDS encompasses various medical conditions such as infections, physical trauma, pancreatitis, pneumonia, aspiration incidents, and extensive blood transfusions, with infections being the predominant cause that is responsible for nearly 40% of all ARDS cases. Globally, ARDS occurs at rates ranging from 7.2 to 34 cases per 100,000 person-years, with a mortality rate between 26 and 35% (Huppert et al., 2019; Yıldırım et al., 2021). In United States ARDS leads to approximately 75,000 deaths annually, affecting around 3 million individuals worldwide. It constitutes 10% of all intensive care unit (ICU) admissions and impacts 23% of ICU patients requiring mechanical ventilation (Huppert et al., 2019).

The pathogenesis of ARDS primarily stems from alveolar injury and increased capillary permeability affecting both the pulmonary endothelium and alveolar epithelial surface. This disruption results in the formation of hyaline membranes and the accumulation of protein-rich fluid within the pulmonary interstitium potentially compromising surfactant molecules, alveolar stability, and gas exchange (Hu et al., 2022). The activation of cytokines and the release of pro-inflammatory mediators exacerbate lung injury and sustain inflammation (Hu et al., 2022). The early phase of ARDS clinically manifests as significant hypoxemia and reduced lung compliance.

A potential association exists between gut microbiota (GM) dysbiosis (GMD) and the initiation and progression of ARDS (Dickson, 2015). The GMD may compromise intestinal barrier function allowing bacteria and toxins to enter the circulatory and lymphatic systems infecting lungs and triggering ARDS. The GM produced metabolites can directly or indirectly induce lung inflammation and GM’s interaction with the immune system influences immune cell activity and inflammatory responses (Sultan et al., 2021). Certain GM bacterial species produce inflammatory factors (IFs) which can influence intestinal epithelial and immune cells (Malesza et al., 2021). The GMD can lead to detrimental changes in intestinal mucosa increasing its permeability and allowing IFs easier access to the bloodstream provoking systemic inflammation (Tilg et al., 2020). Moreover, IFs can impact the GM’s structure and function fostering a self-perpetuating inflammatory cycle (Strati et al., 2022). The GMD negatively affects the immune system heightening inflammatory responses and contributing to ARDS development (Li et al., 2021). The GMD may also influence the lung microbiome contributing to lung inflammation and ARDS. However, the intricate relationship between GM and ARDS remains not entirely understood. Mendelian randomization (MR) employs single nucleotide polymorphisms (SNPs) linked to a specific risk factor as instrumental variables (IVs) to investigate causal relationships between the risk factor and a particular disease (Bowden and Holmes, 2019). Genetic variation occurring from zygote formation engenders considerable variability before disease onset, persisting throughout life and potentially allowing MR studies to bypass biases or confounding factors.

This study aimed to explore the relationship between GM, IFs and ARDS by conducting MR analysis using publicly available summary-level data from genome-wide association studies (GWAS).

## 2 Materials and methods

### 2.1 GWAS data

The GM GWAS data was obtained from MiBioGen study^1^ represent the most extensive multi-ethnic analysis of GM to date. This study, encompassing 16S fecal microbiota data (n = 340) and genotype data (*n* = 13,263) from 16 cohorts (*n* = 24,000) aimed to explore the correlation between GM and human health. The findings revealed significant variability in human GM across different regions, ethnicities and age groups. Moreover, GWAS data on 41 IFs were acquired from a cytokine-related GWAS meta-analysis involving three independent cohorts, including the Young Finns Cardiovascular Risk Study (Suhre et al., 2017; Folkersen et al., 2020). To investigate the potential causal relationship between IFs and ARDS, GWAS data for IFs were extracted from meta-analysis datasets from two cytokine-centric GWAS investigations. Initially, 41 cytokine-related factors compiled by Suhre K were gathered, supplemented by additional cytokine factors collated by Folkersen L. Furthermore, the present study utilized ARDS GWAS data from the Finngen database—a Finnish genetic resource integrating genotypic, phenotypic, diagnostic, and prescription information. This platform primarily focuses on identifying gene-disease associations to drive advancements in disease management. It also provides data analysis tools facilitating global collaboration and knowledge sharing. This study analyzed 216,363 samples (inclusive of 16,380,461 SNPs) from Finngen to investigate ARDS. All exposure and outcome data can be obtained online at IEU.^2^

### 2.2 Selection of IVs

Bacterial classification and analyses were systematically conducted across five taxonomic levels. The inclusion criteria for IVs in this study were meticulously restricted to ensure the precision and validity of causal relationships between GM and the risk of ARDS development. This involved selecting SNPs with a *p*-value <1e-05 as IVs for both exposure and outcome in MR studies. Furthermore, the Two Sample MR R package was utilized with parameters r^2^ = 0.001 and kb = 10,000 to establish IV independence and minimize the potential impact of linkage disequilibrium that could disrupt the random allele assignment process. The SNPs with a minor allele frequency < 0.01 and those exerting a greater influence on outcomes than exposure was subsequently excluded. Furthermore, PhenoScanner V2 was applied to verify that the phenotypes of all SNPs used as IVs were unrelated to the outcomes ensuring adherence to the no-horizontal-pleiotropy assumption in MR analysis (Kamat et al., 2019).

### 2.3 Statistical analysis

Multiple causality analysis models were applied in this study. The inverse-variance weighted (IVW) model and the MR-Egger method (Birney, 2022) were used for analyzing samples with multiple SNPs whereas, Wald ratio test was applied for samples with only one SNP (Ference et al., 2021).

For sensitivity analyses, heterogeneity was assessed using the Cochran Q method. In case of evident heterogeneity (*p* < 0.05) MR-Egger regression analysis was applied to evaluate potential pleiotropic effects of the SNPs used as IVs. In MR-Egger regression the intercept term indicated directed horizontal pleiotropy at *p* < 0.05. The MR-PRESSO methodology was employed to validate MR results. Its use extended to scrutinizing whether the IVs were associated with outliers and assessing the prevalence of horizontal pleiotropy within the global test scope. When pleiotropy was identified, the suggested approach involved excluding outliers, followed by a reiteration of MR and MR-PRESSO analyses. The main objective was to ensure that the *p*-values obtained with both Egger’s intercept and the global test ascertained through MR-PRESSO exceeded the threshold of 0.05 (Verbanck et al., 2018). Additionally, a leave-one-out analysis was utilized to gauge any potential influence of individual SNPs on the outcomes of each MR study. To visualize MR methodologies’ results clearly and concisely, the TwoSampleMR package was used. This facilitated the generation of scatter plots illustrating the overall correlation between exposure and the resulting outcomes. It also produced funnel plots reflecting the symmetric distribution of IVs, in this case, SNPs. Furthermore, forest plots were generated to depict both individual effects and collective exposure-related outcomes’ benefits. The leave-one-out plots demonstrated the stability of outcomes following the removal of specific SNPs. All statistical analyses in this study were conducted using the R package in the R language application (v4.2.1).

## 3 Results

### 3.1 MR analysis of GM and ARDS

**Figure 1:**
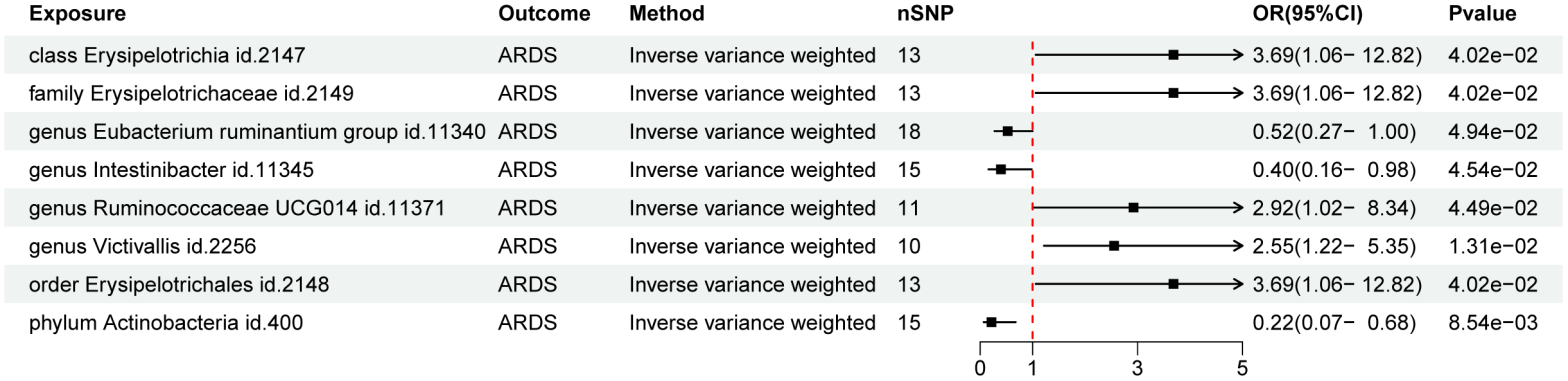
Forest map of the Mendelian randomization analysis of gut microbiota and ARDS.

The preliminary findings suggested that 8 of the 211 gut bacterial taxa had a causal association with ARDS (Figure 1). The results of IVW analysis of these 8 bacterial taxa were as follows: phylum *Actinobacteria* (id.400; *p* = 8.54E-03; odds ratio [OR] 95% confidence interval [CI] = 0.22 [0.07, 0.68]), order *Erysipelotrichales* (id. 2,148; *p* = 4.02E-02; OR [95% CI] = 3.69 [1.06, 12.82]), genus *Victivallis* (id.2256; *p* = 1.31E-02; OR [95% CI] = 2.55 [1.22, 5.35]), genus *Ruminococcaceae* UCG014 (id.11371; *p* = 4.49E-02; OR [95% CI] = 2.92 [1.02, 8.34]), genus *Intestinibacter* (id.11345; *p* = 4.54E-02; OR [95% CI] = 0.40 [0.16, 0.98]), genus *Eubacterium ruminantium* group (id.11340; *p* = 4.94E-02; OR [95% CI] = 0.52 [0.27, 0.99]), family *Erysipelotrichaceae* (id.2149; *p* = 4.02E-02; OR [95% CI] = 3.69 [1.06, 12.82]), and class *Erysipelotrichia* (id.2147; *p* = 4.02E-02; OR [95% CI] = 3.69 [1.06, 12.82]). Among them phylum *Actinobacteria* (id. 400) and genus *Intestinibacter* (id. 11,345) were negatively associated with the risk of ARDS development whereas, the other taxa were positively associated with the risk of ARDS development suggesting that their abundance in GM may lead to ARDS development. Detailed information on MR analysis of GM and ARDS is provided in the Supplementary material.

### 3.2 MR analysis of IFs and ARDS

**Figure 2:**
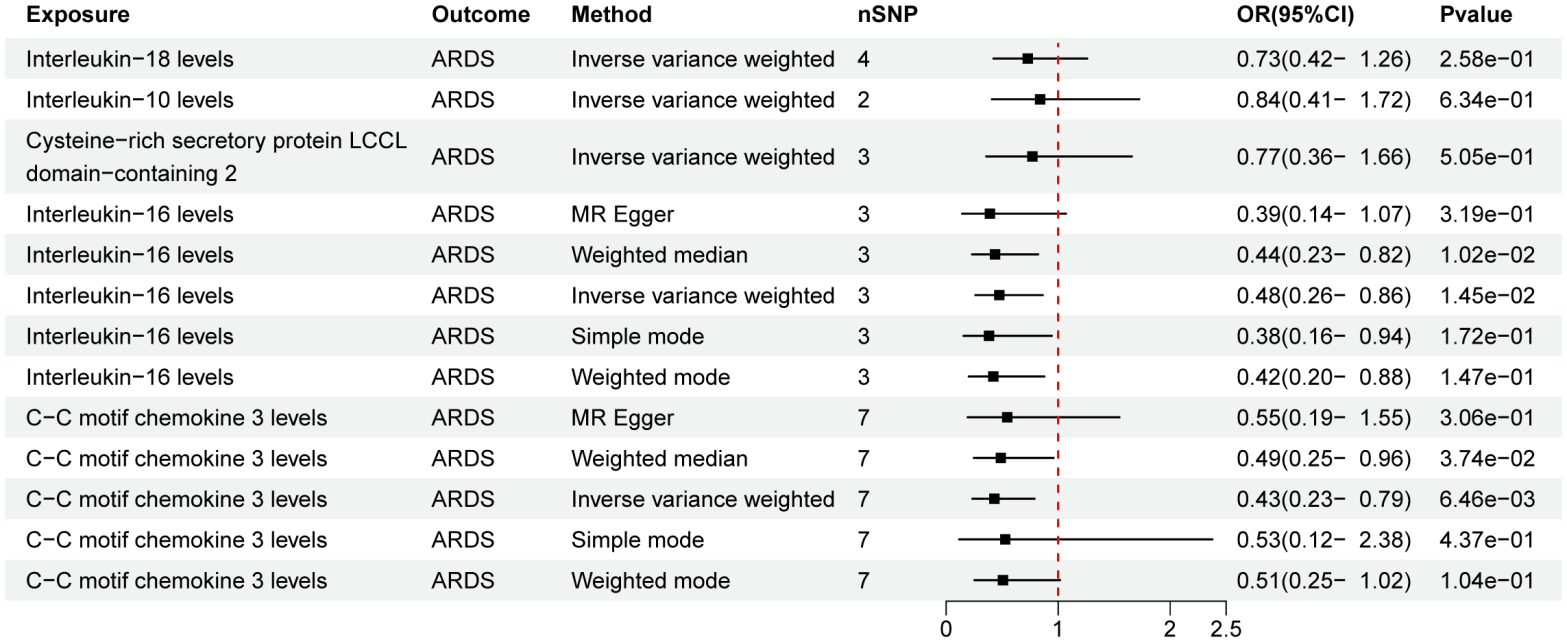
Forest map of the Mendelian randomization analysis of inflammatory factors and ARDS.

Out of the 41 IFs studied only 2 exhibited a potential causal relationship with ARDS (Figure 2). The results of IVW analysis of the 2 IFs indicated significant associations for IL-16 levels (*p* = 1.45E-02; OR [95% CI] = 0.48 [0.26, 0.86]) and C-C motif chemokine 3 levels (CCL3, *p* = 6.46E-03; OR [95% CI] = 0.43 [0.23, 0.79]).

### 3.3 MR analysis of GM and IFs

**Figure 3:**
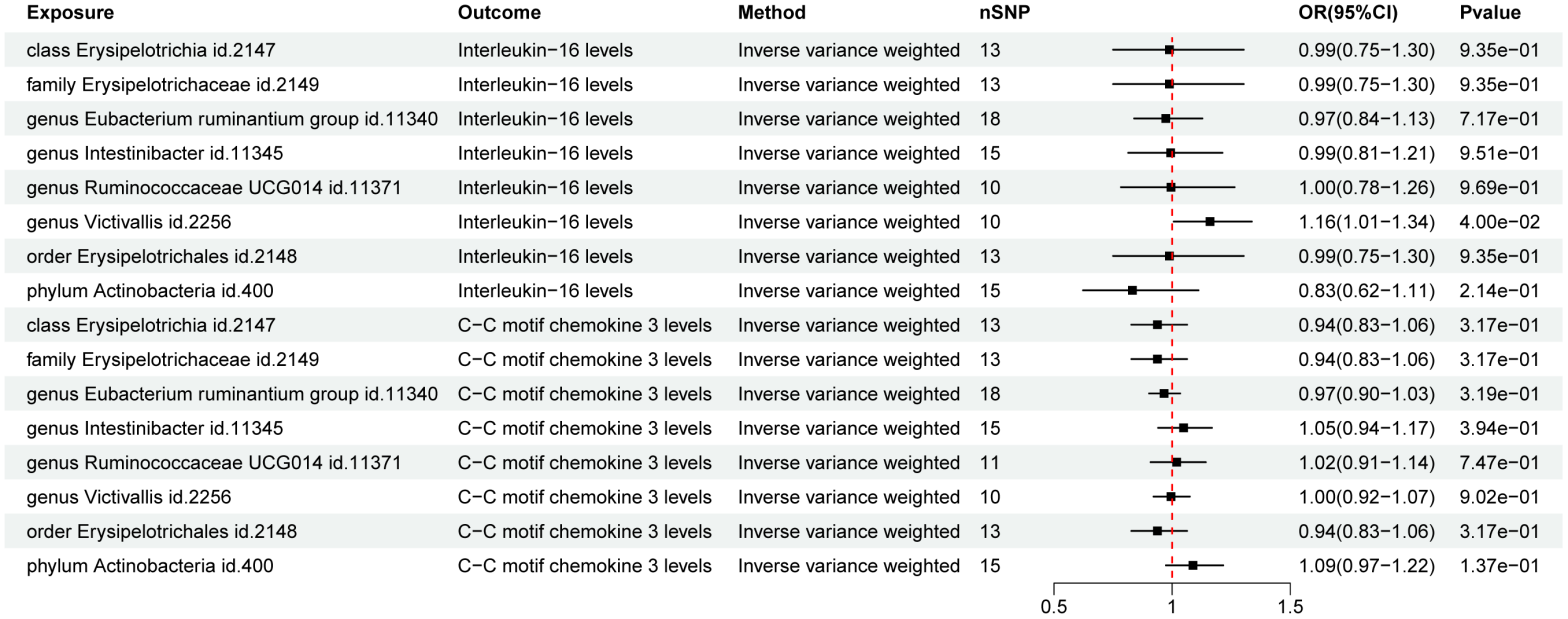
Forest map of the Mendelian randomization analysis of gut microbiota and inflammatory factors.

The MR analysis of GM and IFs aimed to unveil the role of IFs in the relationship between GM and ARDS. The results of IVW analysis revealed a potential positive correlation between genus *Victivallis* (id. 2,256) and IL-16 (*p* = 4.00E-02; OR [95% CI] = 1.16 [1.01, 1.34]) whereas, the other taxa did not exhibit potential relationships with the IFs (Figure 3).

**Figure 4:**
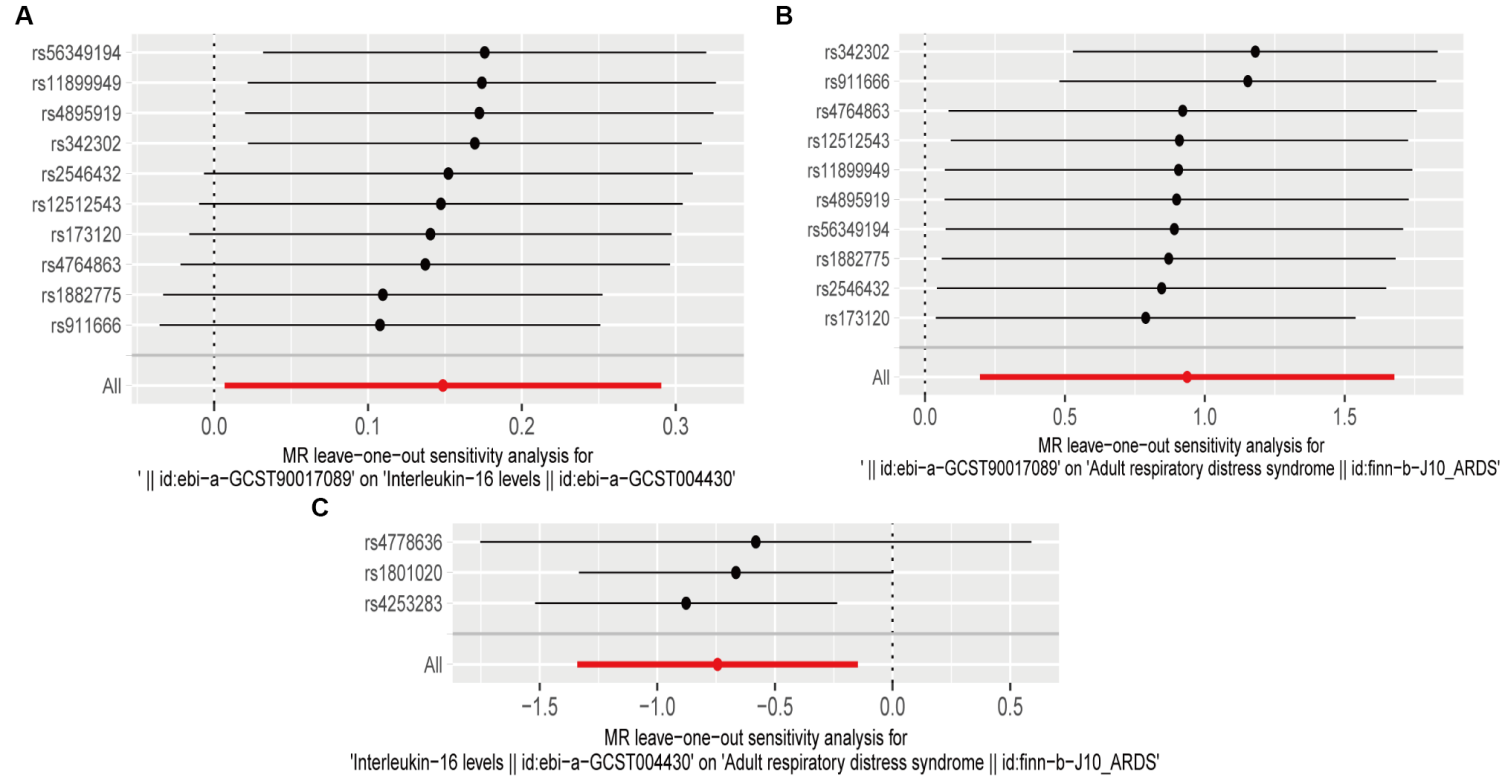
Leave-one-out plots **(A)** leave-one-out plot of genus *Victivallis* id.2256 and interleukin (IL)-16; **(B)** leave-one-out plot of genus *Victivallis* id.2256 and acute respiratory distress syndrome (ARDS); and **(C)** leave-one-out plot of IL-16 and ARDS.

A sensitivity analysis was conducted to validate our results. Both heterogeneity and multiplicity tests showed a *value of p* of <0.05, indicating the absence of abnormalities. Furthermore, the findings of the present study remained robust and consistent in subsequent leave-one-out analyses (Figure 4). Detailed results of the sensitivity analysis are presented in the Supplementary material.

## 4 Discussion

ARDS is a severe lung disease characterized by inflammation and injury to the alveoli and alveolar capillaries (Yıldırım et al., 2021). Infection and inflammation are the major factors for ARDS (Odeyemi et al., 2019; Meyer et al., 2021). Recent studies have unveiled a strong correlation between GMD and the onset of ARDS. The GM is the largest microbial community in body and plays a vital role in maintaining gut health and immune function (Dickson et al., 2016). However, critically ill patients may experience significant alterations in both gut and lung microbiota. The lung microbiota of patients with infection and patients with ARDS has an abundance of gut-related microorganisms which may enter the lungs through bacterial translocation from the gut which is facilitated by increased gut and alveolar-capillary permeability (Dickson et al., 2020). Moreover, the lung microbiota of patients on ventilators also exhibits an enrichment of gut-related microorganisms which are associated with ARDS occurrence. These findings suggest that GMD is a crucial factor in the development and progression of ARDS (Mustansir Dawoodbhoy et al., 2021). The GMD potentially contributes to ARDS development and progression through various mechanisms. First, GMD may lead to the disruption of intestinal barrier function, permitting bacteria and their metabolites to enter the circulatory system, thus affecting immune and inflammatory responses in the lungs (Hu et al., 2023). Second, GMD might prompt the release of intestinal toxins, further activating the inflammatory response and causing inflammation and injury in the lungs. In this study, eight bacterial taxa were identified to have a potential association with ARDS, including *Actinobacteria* (id. 400), *Erysipelotrichales* (id. 2,148), *Victivallis* (id. 2,256), *Ruminococcaceae* UCG014 (id. 11,371), *Intestinibacter* (id. 11,345), *Eubacterium ruminantium* group (id. 11,340), *Erysipelotrichaceae* (id. 2,149) and *Erysipelotrichia* (id. 2,147).

Inflammation plays a pivotal role in driving the occurrence and progression of ARDS. The initiation of ARDS is typically linked to pulmonary infections, trauma, and pancreatitis all causing significant damage to the lung tissues and triggering an inflammatory response. This response leads to the accumulation and activation of immune cells such as neutrophils and monocytes which are responsible for releasing inflammatory mediators such as cytokines and chemokines which further exacerbate the inflammatory response (Alghetaa et al., 2021). In the early stages of ARDS, this response disrupts the alveolar and vascular endothelial barriers leading to lung tissue fluid accumulation and pulmonary edema formation (Yin et al., 2017). Furthermore, inflammation contributes to fibrosis within lung tissue compromising pulmonary function. Moreover, it may lead to a cytokine storm which is characterized by extensive activation immune cells as well as excessive release of cytokines. This pathological event frequently causes immune dysregulation, inflammation and pulmonary fibrosis which leads to escalating the severity of inflammation and the resultant lung tissue damage (Martin-Loeches et al., 2020; Siwicka-Gieroba and Czarko-Wicha, 2020). Furthermore, in patients with ARDS peripheral blood mononuclear cells and natural killer cells highly express genes closely associated with the inflammatory response (Zheng et al., 2023; Zou et al., 2023) including genes related to pro-inflammatory cytokines and inflammation-related signaling pathways.

This study suggests that IL-16 and CCL3 play significant roles related to the pathogenesis of ARDS. The IL-16 (a cytokine) and CCL3 (a chemokine, also known as macrophage inflammatory protein-1α) have consistently showed significantly upregulated expression in patients with ARDS (Cruikshank et al., 2000; Houshmandfar et al., 2021; Kono et al., 2023). Increased levels of IL-16 and CCL3 lead to inflammatory cell aggregation and intensifying lung inflammation. Moreover, they can prompt the release of cytokines such as IL-1β, IL-6 and TNF-α resultantly exacerbating lung inflammation (Kono et al., 2023). High IL-16 expression has been linked to increased severity and poor prognosis in ARDS cases (Tsagkaris et al., 2022; Kono et al., 2023). The CCL3 activates inflammatory cells, such as monocytes and lymphocytes and facilitates their entry into the lungs contributing to inflammatory response (Houshmandfar et al., 2021). The CCL3 is also reported to promotes the adhesion and migration of inflammatory cells worsening lung injury (Baier et al., 2004).

This study not only uncovers critical connections between IFs such as IL-16, CCL3, and ARDS but also establishes a link between these factors and the role of GM in ARDS progression. The findings offer a comprehensive understanding related to the pathogenesis of disease. The MR analysis was applied to the publicly available GWAS data for exploring the intricate relationship among GM, IFs, and ARDS. Our findings show that the phylum *Actinobacteria* (id. 400) and the genus *Intestinibacter* (id. 11,345) are negatively correlated with the risk of ARDS development whereas, other bacterial groups exhibit positive correlations with the risk of ARDS development. Furthermore, our results indicate a potential causal relationship between IL-16, CCL3 and ARDS. Further analysis between GM and IFs suggests a possible positive correlation between the genus *Victivallis* and IL-16.

Nevertheless, this initial investigation comes with certain constraints as our analysis is dependent on publicly accessible GWAS data which carries certain limitations and biases. Moreover, the MR technique aids in evaluating causal associations and it may not completely eradicate the influence of confounding variables. Therefore, further research is imperative to corroborate and enhance our comprehension of the association between GM and ARDS. This extended exploration will not only fortify the evidence but also contribute to a more thorough understanding of the mechanisms that interconnect these factors.

## 5 Conclusion

Mendelian randomization (MR) analysis unveiled the potential significant contributions of gut microbiota (GM) and inflammatory factors (IFs) to both the initiation and advancement of acute respiratory distress syndrome (ARDS). The analysis identified 8 out of 211 gut bacterial taxa demonstrating potential causal associations with ARDS. *Actinobacteria* (id. 400) and *Intestinibacter* (id. 11,345) exhibited a negative association with the risk of ARDS development whereas, *Erysipelotrichales* (id. 2,148), *Victivallis* (id. 2,256), *Ruminococcaceae* UCG014 (id. 11,371), *Eubacterium ruminantium* group (id. 11,340), *Erysipelotrichaceae* (id. 2,149) and *Erysipelotrichia* (id. 2,147) showed a positive association with the risk of ARDS development. Furthermore, MR analysis examining IFs and ARDS indicated potential causal links with interleukin-16 (IL-16) and C-C motif chemokine 3 (CCL3). Further exploration uncovered a potential positive correlation between the genus Victivallis (id. 2,256) and IL-16. These findings suggest that GM and IFs may influence immune and inflammatory responses, thereby impacting the development and progression of ARDS.

## Data availability statement

The datasets presented in this study can be found in online repositories. The names of the repository/repositories and accession number(s) can be found in the article/Supplementary material.

## Author contributions

JM: Writing – original draft. ZZ: Conceptualization, Writing – Original Draft. YY: Formal analysis, Investigation, Writing – original draft. KA: Writing – Review & Editing. LH: Supervision, Writing – review & editing. LL: Supervision, Writing – review & editing.

## Footnotes

^1^https://mibiogen.ARDSc.rug.nl/

^2^https://gwas.mrcieu.ac.uk/
